# Anti-Inflammatory microRNAs for Treating Inflammatory Skin Diseases

**DOI:** 10.3390/biom12081072

**Published:** 2022-08-03

**Authors:** Shih-Chun Yang, Ahmed Alalaiwe, Zih-Chan Lin, Yu-Chih Lin, Ibrahim A. Aljuffali, Jia-You Fang

**Affiliations:** 1 Department of Microbiology, Soochow University, Taipei 111, Taiwan; scyang@scu.edu.tw; 2 Department of Pharmaceutics, College of Pharmacy, Prince Sattam Bin Abdulaziz University, Al Kharj 11942, Saudi Arabia; alalaiwe@gmail.com; 3 Pharmaceutics Laboratory, Graduate Institute of Natural Products, Chang Gung University, Kweishan, Taoyuan 333, Taiwan; sevenaurora@gmail.com; 4 Department of Environmental Engineering and Health, Yuanpei University, Hsinchu 300, Taiwan; yuchihlin@mail.ypu.edu.tw; 5 Department of Pharmaceutics, College of Pharmacy, King Saud University, Riyadh 11362, Saudi Arabia; ialjuffali@ksu.edu.sa; 6 Department of Anesthesiology, Chang Gung Memorial Hospital, Kweishan, Taoyuan 333, Taiwan; 7 Research Center for Food and Cosmetic Safety and Research Center for Chinese Herbal Medicine, Chang Gung University of Science and Technology, Kweishan, Taoyuan 333, Taiwan

**Keywords:** microRNA, skin, anti-inflammation, inflammatory disease, keratinocyte

## Abstract

Skin inflammation occurs due to immune dysregulation because of internal disorders, infections, and allergic reactions. The inflammation of the skin is a major sign of chronic autoimmune inflammatory diseases, such as psoriasis, atopic dermatitis (AD), and lupus erythematosus. Although there are many therapies for treating these cutaneous inflammation diseases, their recurrence rates are high due to incomplete resolution. MicroRNA (miRNA) plays a critical role in skin inflammation by regulating the expression of protein-coding genes at the posttranscriptional level during pathogenesis and homeostasis maintenance. Some miRNAs possess anti-inflammatory features, which are beneficial for mitigating the inflammatory response. miRNAs that are reduced in inflammatory skin diseases can be supplied transiently using miRNA mimics and agomir. miRNA-based therapies that can target multiple genes in a given pathway are potential candidates for the treatment of skin inflammation. This review article offers an overview of the function of miRNA in skin inflammation regulation, with a focus on psoriasis, AD, and cutaneous wounds. Some bioactive molecules can target and modulate miRNAs to achieve the objective of inflammation suppression. This review also reports the anti-inflammatory efficacy of these molecules through modulating miRNA expression. The main limitations of miRNA-based therapies are rapid biodegradation and poor skin and cell penetration. Consideration was given to improving these drawbacks using the approaches of cell-penetrating peptides (CPPs), nanocarriers, exosomes, and low-frequency ultrasound. A formulation design for successful miRNA delivery into skin and target cells is also described in this review. The possible use of miRNAs as biomarkers and therapeutic modalities could open a novel opportunity for the diagnosis and treatment of inflammation-associated skin diseases.

## 1. Introduction

Ribonucleic acids (RNAs) are able to regulate gene expression at the transcriptional, posttranscriptional, and epigenetic stages. Noncoding RNAs comprise a major portion of the human transcriptome. These functional RNAs include ribosomal RNA (rRNA), long noncoding RNA (lncRNA), small interfering RNA (siRNA), piwi-interacting RNA (piRNA), and microRNA (miRNA) [1]. Among these, miRNA is a small and highly conserved noncoding RNA sequence containing 19–25 nucleotides. This single-stranded RNA can regulate the expression of protein-coding genes at the posttranscriptional level to join the maintenance of correct cell homeostasis [2]. Since the discovery of miRNA in 1993, 5000–10,000 miRNAs have been found in mammals. miRNAs comprise 1–5% of all genes in the human genome [3]. Approximately 20–60% of protein-coding genes are regulated by miRNAs. miRNAs participate in cell development, morphogenesis, proliferation, apoptosis, differentiation, immune regulation, and wound healing [4]. Under the condition of disease, miRNAs can change to induce altered gene expressions, leading to aberrant phenotypes. On the other hand, they also predominate the protective capacity by reestablishing cell homeostasis [5]. The balance of miRNA plays a key role in the correct functioning of cell physiology. The exploration of miRNA has advanced the development of molecular biology, bioinformatics, and translational investigation.

**Figure 1:**
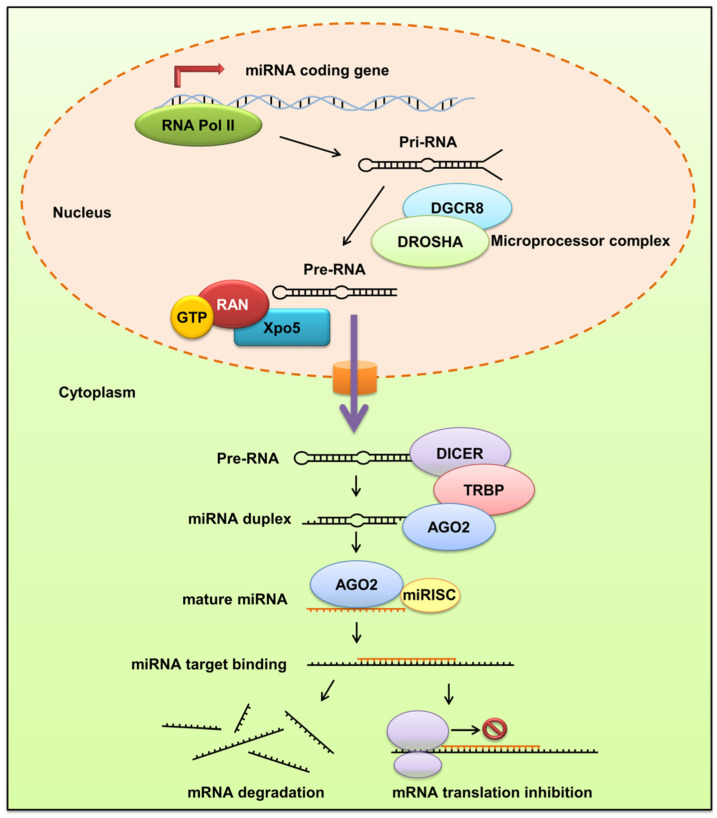
The biogenesis and gene expression of miRNA in cells.

Most miRNAs are transcribed from deoxyribonucleic acid (DNA) sequences in the nucleus by RNA polymerases [6]. Drosha is a member of the RNase III family that cleaves the primary miRNA (pri-miRNA) to generate a 70-nucleotide precursor miRNA (pre-miRNA). The pre-miRNA is transported to the cytoplasm by exportin-5 and is then processed by the RNase III endonuclease dicer to produce mature miRNA. The mature miRNA is loaded onto the RNA-induced silencing complex (RISC) as guided by the Argonaute (AGO) family of proteins for binding to the 3′-untranslated region (3′UTR) of the target messenger RNA (mRNA) [7]. This can result in the translation-suppression or degradation of the target mRNA. The biogenesis of miRNA and its impact on mRNA are illustrated in Figure 1. miRNA dysregulation is involved in a broad range of diseases, including developmental abnormalities, cancer, metabolic disease, autoimmune disorders, and cardiovascular dysfunction [8]. The modulation of disease-associated miRNAs is beneficial for the targeted therapy of several diseases. Different from the conventional approaches of turning off specific targets, miRNAs exert a biological function by tuning protein-coding genes. miRNA expression modulation has some advantages. Introducing siRNA into cells can reduce the expression of specific genes. However, an unpredicted effect of siRNA on the cells via off-target effects may happen. In the case of miRNA, one exogenous miRNA can modulate several genes that often act in the same biological pathway. In addition, the action of miRNA is designed by nature itself. The intervention of miRNA-based therapies usually causes limited toxicity or adverse impacts [9]. Besides its action in cell interiors, miRNA can be released into plasma, tissue fluid, urine, and milk. miRNA is protected by exosomes or combined with high-density lipoproteins to avoid enzymatic degradation in the plasma [10]. The exosomal miRNA secreted by the cells exerts a vital role in cell-to-cell communication. It is capable of penetrating neighboring cells and can control the expression of genes [11]. In the past few years, several miRNA-based therapeutics have been developed and are currently in different phases of clinical trials [12]. Clinical trials of numerous miRNAs have shown positive results in their initial phases. Some miRNA molecules are in different stages of clinical trials, including the treatment of hepatitis C virus infection (phase II), mycosis fungoides (phase II), polycystic kidney disease (phase I), cutaneous T cell lymphoma (phase I), hepatocellular carcinoma (phase I), malignant pleural mesothelioma (phase I), ischemia (phase I), heart failure (phase I), and idiopathic pulmonary fibrosis (phase I) [13,14]. Although a future therapeutic application of miRNAs is appealing, there are still great practical difficulties to overcome, such as the identification of proper administration routes, the control of in-body stability, the targeting of specific cells, and the attaining of the intended intracellular effects.

Inflammation is a protective strategy of the cells to neutralize the stimuli-including pathogens, toxins, irritants, mechanical stress, and allergens. However, inappropriate inflammation can cause tissue damage. The activation of excessive inflammation is detected by sensors, such as toll-like receptors (TLRs), which are found in macrophages, dendritic cells, and mast cells [15]. This activation induces the production of proinflammatory mediators, including cytokines and chemokines. Apart from the role of regulating cell-normalization processes, miRNA exhibits disturbed expression in inflammatory and autoimmune diseases. Altered miRNA expression is associated with inflammatory signaling, increased cytokine release, and preservation of the vicious cycle in autoimmunity [16]. On the other hand, miRNA can act as part of a negative regulatory loop to keep inflammation in check by elevating anti-inflammatory mediator generation for the return to homeostasis [17]. As the largest organ of the human body, the skin requires a large amount of highly regulated miRNAs for its development and morphogenesis. miRNAs are involved in skin immunity, cell proliferation, aging, pigmentation, wound healing, and cutaneous microbiomes [18,19]. miRNAs also play a role in skin cancers, inflammatory skin diseases, and autoimmune skin disorders. Thus, miRNAs can be biomarkers for skin diseases because of the different expression levels of miRNAs between lesional and healthy skin. miRNAs can also be cell-specific markers for skin disease diagnosis and prognosis. The treatment efficacy and the therapeutic outcome can also be evaluated by changes in the miRNA levels. For example, psoriasis patients show higher levels of miR-125b, miR-146a, miR-203, and miR-223 in serum as compared with healthy subjects [20,21]. It has also been observed that miR-424 is largely detected in the hair shafts of psoriasis patients as compared with normal subjects and those with atopic dermatitis (AD) [22]. The serum of pediatric AD patients shows upregulated miR-203 and miR-483-5p levels compared with healthy groups [23]. miR-194-5p is a useful biomarker for AD diagnosis because of its downregulation in the plasma of AD patients [24]. Another AD biomarker, detected in peripheral CD4+ T cells, is the significant elevation of miR-155 in AD patients [25].

To date, no successful therapy has been found to completely cure autoimmune and inflammatory skin diseases and prevent their recurrence. Current long-term therapy is also difficult because of the inefficiency after prolonged application and the adverse effects of the treatments. There is an emerging need to develop efficient therapeutic strategies to manage chronic inflammatory skin diseases. miRNA-based therapies have become potential candidates for treating inflammatory skin diseases over the last decade. The dermatological or cosmeceutical application of active ingredients to regulate miRNA expression for treating skin diseases has also been largely recognized [26]. There are two approaches for employing miRNA as a gene modulator: miRNA inhibitors/antagomirs, and miRNA mimics/agomirs. The miRNA inhibitor/antagomir approach is utilized for the aberrantly expressed miRNAs that are upregulated in diseases, while anti-miRNA or miRNA inhibitors specifically bind to endogenous mature miRNA, thus preventing targeted miRNA expression [27]. In contrast, miRNAs that are reduced in diseases can be supplied transiently using miRNA mimics and agomirs. Mimics are chemically designed and synthesized to simulate endogenous miRNAs. This review focuses on miRNA mimics that exert anti-inflammatory activity for treating cutaneous inflammation-related diseases, including psoriasis, AD, lupus, skin wounds, and skin aging. Natural or synthetic actives and drugs that can modulate miRNAs for mitigating skin inflammation are also discussed in this study. Most naked miRNAs can usually neither permeate the skin nor facilely cross the cell membrane because of their large size and negative charge [28]. A delivery system is, therefore, required for miRNA administration. This study additionally highlights the emerging approach of formulating designs for miRNAs to achieve successful and efficient delivery into the nidus or target cells.

## 2. miRNAs and Inflammation

**Figure 2:**
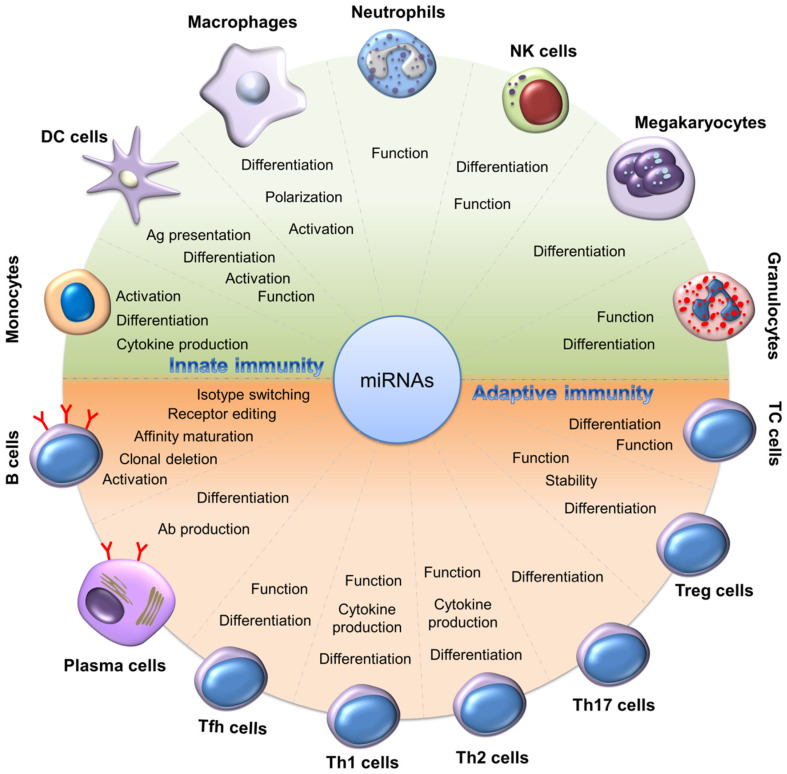
The function of miRNA in the regulation of innate or adaptive immune responses in different cells.

Inflammation is a complicated pathophysiological cascade of the response to infection or injury. The mechanism of inflammation is closely associated with many human diseases. The magnitude and network of pro- and anti-inflammatory factors affect the development and progression of various diseases. Inflammation regulation can be governed by the coordinated control of gene expression in participating immune cells and systems [29]. miRNA is the key gene regulator to achieve inflammation control. Anti-inflammatory miRNAs are fine-tuned signaling regulators that allow the resolution and prevention of inflammatory reactions in immune cells [30]. miRNA has an extensive spectrum of biofunctions for inflammation regulation in immune cells (Figure 2). miRNAs can either enhance or inhibit inflammation, depending on the target mRNAs. The immune system employs multiple miRNAs to manage the functional capacity for constructing a balance between activation and suppression. Innate defense pathway stimulation, such as that found in TLR signaling, contributes to the altered expression of miRNAs that modulate inflammatory genes. Some anti-inflammatory miRNAs modulate the translation of transcripts, leading to a reduction in the immunomodulating factor levels for inhibiting or regulating inflammatory responses [31].

Some miRNAs inhibit multiple target genes involved in inflammation-related signaling. The manipulation of the miRNA expression level offers an applicable therapy against inflammatory diseases. The targeting of the inflammatory response through miRNA mimics could be an effective treatment. Anti-inflammatory miRNA mimics for inflammation mitigation have been previously reported [17]. These include miR-10a, miR-21, miR-24, miR-106b, miR-124, miR-143, miR-145, miR-146, miR-155, and miR-375. These miRNAs can be a negative regulator of inflammation by targeting several inflammation-related pathways, such as TLR, signal transducer and activator of transcription (STAT), nuclear factor-κB (NF-κB), tumor necrosis factor receptor (TNFR)-associated factor 6 (TRAF6), and Janus kinase (JAK). The overexpression of anti-inflammatory miRNAs in turn abrogates the production of proinflammatory cytokines and chemokines in the immune cells, resulting in the attenuation of the inflammatory response [32]. Numerous miRNAs function in the downregulation of inflammatory pathways. For biological consideration, this is an ideal and precise coordination system to control inflammation. As inflammation is initiated, the fast transcriptional upregulation of the proinflammatory mediators occurs. At the same time, the expression of some miRNAs is initiated by the same transcription. These miRNAs either restrain the expression of the positive signaling proteins or inhibit the same pathway [33]. Altered miRNA expression and supplementary anti-inflammatory miRNA mimics have been successfully used to treat inflammatory and immunological skin disorders. This can open a new field to explore pathogenesis, develop novel biomarkers for diagnosis, and design mechanism-driven therapeutic approaches.

## 3. miRNAs for Treating Inflammatory Skin Diseases

### 3.1. Psoriasis

**Figure 3:**
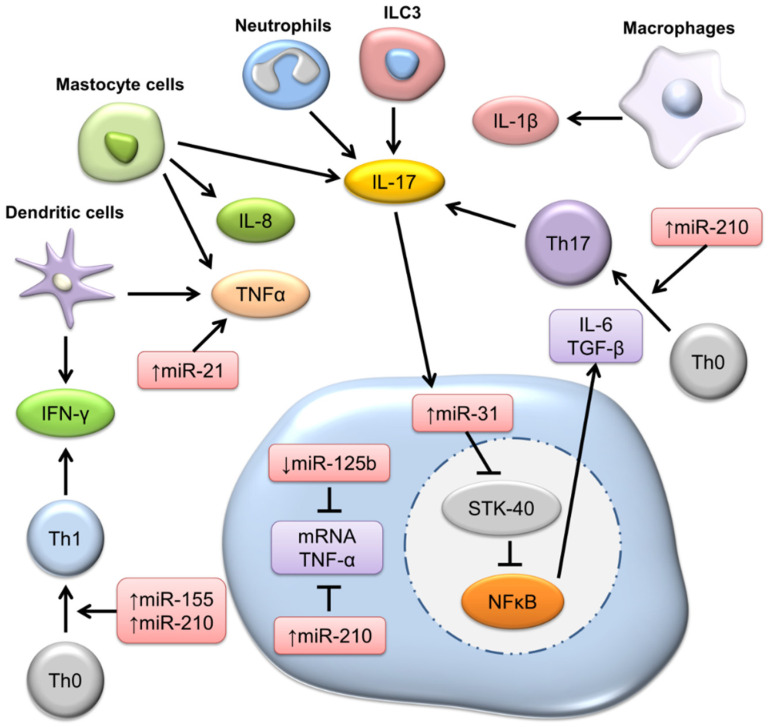
The role of miRNA in the regulation of T cell differentiation and cytokine production in psoriasis.

Psoriasis is a chronic autoimmune skin disease delineated by epidermal hyperplasia and inflammatory cell infiltration. The worldwide prevalence of psoriasis is 2–3%, and patients with severe psoriasis have a shortened life expectancy [34]. Both genetic and environmental factors, in association with irregular immune systems, are considered to be involved in psoriatic pathogenesis. Keratinocytes and immune cells are responsible for the production of proinflammatory mediators after activation, leading to keratinocyte proliferation and amplification loops in psoriatic lesions [35]. The suppression of hyperproliferation and inflammation is a target for antipsoriatic therapies [36]. Psoriasis is strongly dependent on genomic variation. A growing number of psoriasis-susceptible genes involved in immunity and keratinocyte function have been discovered [37]. The elucidation of these genes is essential to understand the pathogenetic mechanisms of psoriasis. miRNA dysregulation has been detected in psoriasis patients. Since the first discovery of altered miRNA expression in psoriasis [38], more than 250 miRNAs have been found to be differentially expressed in the skin and blood of psoriasis patients [39]. miRNAs have the potential to predominate the proliferation, apoptosis, differentiation, and proinflammatory mediator production of keratinocytes, as well as the activation of immune cells [40]. Increasing evidence highlights the successful use of miRNAs as psoriasis biomarkers for diagnosis, prognosis, and therapeutic response monitoring (Figure 3).

It has been proven that miR-21, miR-31, miR-146a, miR-155, and miR-203 are greatly upregulated in the lesional skin of psoriasis patients [41], among which miR-21-3p and miR-21-5p play a crucial role in epidermal inflammation. miR-31 functions in the positive vicious loop in psoriatic keratinocytes through proliferative, differentiative, and inflammatory mechanisms [42]. On the other hand, miR-146a and miR-203 are known to hold negative potential in epidermal inflammation, participating in the balance of keratinocyte proliferation and differentiation [43]. The overexpression of miR-155 in psoriasis skin reduces loricrin expression in keratinocytes and disrupts the epidermal barrier’s properties [44]. In addition to lesional skin, some miRNAs in the serum of psoriasis patients are increased as compared with healthy patients. The serum levels of miR-33, miR-126, miR-143, and miR-223 are elevated in psoriasis patients, serving as biomarkers for disease severity and therapeutic outcome [45]. Both inflammatory and anti-inflammatory miRNAs are associated with the initiation, development, and maintenance of psoriasis. When treating psoriasis, some miRNA mimics can be administered to patients to relieve symptoms via genomic regulation.

miR-99a is downregulated in psoriatic lesions by targeting Frizzled (FZD)5 and FZD8. The wingless-related integration site (Wnt)/β-catenin axis plays an important role in cell proliferation. This pathway is activated by binding the Wnt ligand to the FZD receptor protein [46]. Shen et al. [47] delivered miR-99a mimics into keratinocytes (HaCaT) to achieve miR-99a overexpression. The miR-99a mimics suppressed keratinocyte proliferation via the reduction of FZDs by about two-fold. The examination of FZD expression in the lesional skin of psoriasis patients exhibited an inverse correlation of miR99a with FZD5 (*p* = 0.018) and FZD8 (*p* = 0.003). miR-125a was found to be intimately related to immunity and inflammation [48]. The quantitative reverse-transcription polymerase chain reaction (RT-qPCR) data from 60 psoriasis patients demonstrated the reduction of miR-125a in lesional skin compared with non-lesional sites [49]. miR-125a was negatively correlated with TNF-α (*p* = 0.001), IL-1β (*p* = 0.014), and IL-17 (*p* = 0.003) in lesional skin. The miR-125a mimic transfection into HaCaT led to the inhibited proliferation and increased apoptosis for abrogating keratinocyte activation.

The miR-146 family consists of miR-146a and miR-146b, which are encoded by genes located on chromosomes 5 and 10, respectively [50]. Srivastava et al. [51] found a protective capability of miR-146a for early psoriasis onset. The genetic deficiencies of miR-146a resulted in exacerbated skin inflammation after imiquimod stimulation in miR-146a^−/−^ mice. Imiquimod is a TLR agonist that induces psoriasiform skin in murine models [52]. The intradermal injection of synthetic miR-146a in wild-type mice bearing psoriasiform dermatitis led to a 14-fold increase in miR-146a expression as compared with the scramble control. This overexpression caused the mitigation of erythema, epidermal thickness, scaling, and neutrophil infiltration. miR-146b can assist miR-146a in the suppression of the inflammatory response in psoriasis [53]. Interferon (IFN)-γ- or TNF-α-stimulated keratinocytes were transfected by miR-146b mimics. The result showed a significant inhibition of IL-1R-associated kinase (IRAK1), fermitin family homolog 1 (FERMT1), IL-8, and chemokine (C-C motif) ligand (CCL)5 after miR-146b treatment. This effect was similar to the result of miR-146a mimic intervention, leading to the hindrance of keratinocyte proliferation. Both miRNAs target similar sets of transcripts. SERPINB2 is a serine protease inhibitor subgroup member of the serpin superfamily. This inhibitor is upregulated under infection and inflammation conditions in macrophages, monocytes, fibroblasts, eosinophils, and keratinocytes [54]. Vaher et al. [55] found that overexpressed SERPINB2 in the psoriatic skin is positively related to psoriasis severity and negatively related to miR-146a/b. Silencing the caspase recruitment domain family member 10 (CARD10) and IRAK, the direct targets of miR-146a/b, reduced SERPINB2 expression in keratinocytes. Thus, miR-146a/b and SERPINB2 coordinately act in the hindrance of psoriasis-associated inflammation.

Tang et al. [56] demonstrated that miR-187 declines in cytokine-activated HaCaT and the lesional skin of psoriasis patients. In their study, the exogenous miR-187 agomir (10 nmol) was intradermally delivered to imiquimod-treated psoriasiform mice to increase the level of miR-187. The overexpression of miR-187 lessened acanthosis and inflammation in the mice, and this effect was due to the hyperproliferation inhibition by targeting CD276. CD276, also known as B7 homolog 3 protein, is an immune checkpoint molecule belonging to the B7-CD28 family [57]. miR-193b-3p is another anti-inflammatory miRNA used to achieve the amelioration of psoriasis. Huang et al. [58] transfected miR-193b-3p mimics in HaCaT and observed suppressed proliferation and NF-κB/STAT3 signaling. The bioinformatic analysis and dual-luciferase reporter assay indicated that miR-193b-3p could diminish keratinocyte activation by directly targeting the Erb-B2 receptor tyrosine kinase 4 (ERBB4). Intradermal injections of miR-193b-3p agomirs into the imiquimod-treated mice dramatically increased miR-193b-3p expression by about six-fold. This overexpression reduced the epidermal thickness from 160 to 50 μm in the psoriasiform skin. miR-203 is largely expressed in keratinocytes to inhibit p63 and suppressors of cytokine signaling (SOCS)3 for regulating cell differentiation [59]. Wang et al. [60] verified the role of miR-203a in inflammation regulation in psoriasis by pcDNA3.1-miR-203 plasmid transfection into HaCaT. The 3′-untranslated region (UTR) of kynureninase was the conserved target area of the miR-203a. The overexpressed miR-203a (by eight-fold) inhibited kynureninase, thereby inhibiting the production of IL-1β in the keratinocytes. The in vivo psoriasiform mouse model also exhibited miR-203a-induced inversed kynureninase expression during the development of psoriatic inflammation.

The transcriptomic profile of clinical psoriasis verified a downregulation of miR-214-3p in psoriatic lesions compared with healthy skin [61]. The TNF inhibitor adalimumab can increase the miR-241-3p levels in the lesional skin of psoriasis patients by 1.7-fold [41]. Zhao et al. [62] demonstrated a negative regulation of forkhead box M1 (FOXM1) by miR-214-3p, inhibiting keratinocyte hyperproliferation. FOXM1 is a proliferation-specific transcription factor belonging to the forkhead family. The intradermal administration of miR-214-3p mimics in imiquimod-induced psoriasis-like mice alleviated the signs of erythema, scales, and epidermal thickness. FOXM1 expression in the lesions was reduced by about two-fold after miR-214-3p application. Liu et al. [63] indicated the downregulation of miR-215-5p in cytokine-stimulated HaCaT and imiquimod-treated skin tissue. The treatment of miR-215-5p agomirs on imiquimod-treated mice decreased the number of Ki67-positive cells in the epidermis. The luciferase assay suggested that miR-215-5p bound to the 3′UTR of dual-specificity tyrosine phosphorylation regulated kinase 1A (DYRK1A) as the direct target. Both cell- and animal-based studies showed that miR-215-5p negatively regulated DYRK1A, inhibiting the downstream pathways of protein kinase B (AKT) and extracellular signal-regulated kinase (ERK). Bian et al. [64] demonstrated that miR-340 reduced IL-17A expression in 293T cells through IL-17A 3′UTR. Imiquimod-stimulated skin inflammation in mice treated with intravenous miR-340 agomir resulted in substantially lower scores for cutaneous redness, scaling, and thickening. The cumulative psoriasis severity score could be reduced from nine to six after agomir treatment. miR-383 functions as a suppressor of tumor progression and cell proliferation [65]. It has been reported that miR-383 can target the 3′UTR of lipocalin 2 (LCN2) and block JAK/STAT activation [66]. Skin cells from imiquimod-treated rats were transfected with miR-383 mimics. Overexpressed miR-383 and decreased LCN2 expression were detected by this transfection. Compared with the control, the miR-383 mimic treatment reduced cell proliferation while increasing cell apoptosis. Ye et al. [67] reported downregulated miR-489-3p expression in psoriasis patients. A further bioinformatic assay and luciferase reporter study indicated the direct targeting of miR-489-3p to TLR4 in keratinocytes. HaCaT cells transfected with miR-489-3p mimics inhibited cell proliferation and TLR4/NF-κB signaling. The TNF-α, IL-1β, IL-22, and IFN-γ levels declined by about two-fold after miR-489-3p transfection. The targets and biological mechanisms of the anti-inflammatory miRNAs for treating psoriasis are summarized in Table 1.

### 3.2. Atopic Dermatitis (AD)

**Figure 4:**
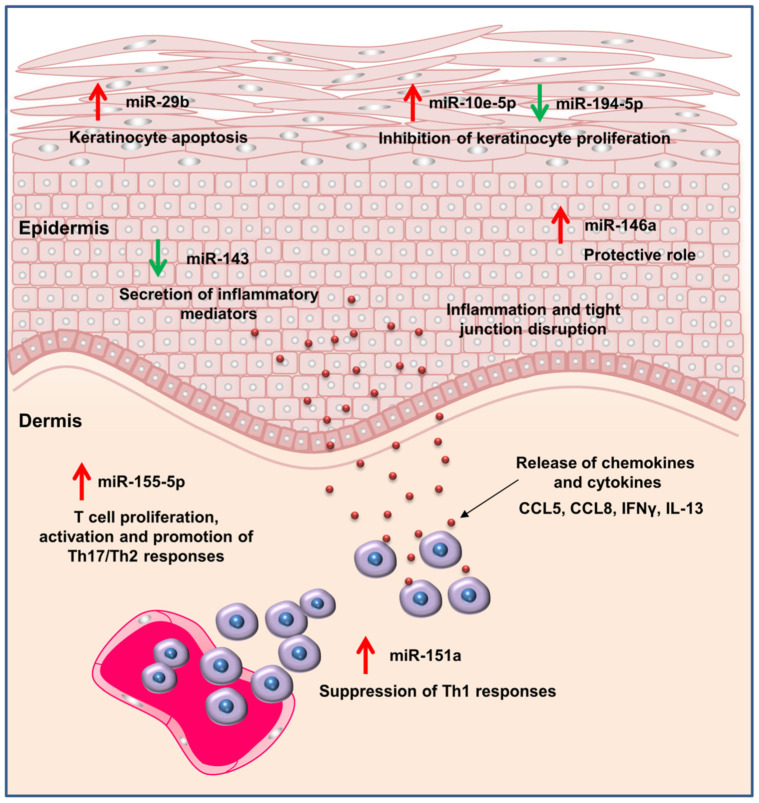
The dysregulation of miRNAs involved in atopic dermatitis and their effect on pathogenesis.

AD is an inflammatory skin disease characterized by erythema, edema, vesicles, and lichenification. The pathogenesis of AD is involved in inflammation dysregulation and response to antigens. AD can be featured by skin barrier dysfunction, skin microbiome alteration, and type 2 immune responses [68]. The increases in immunoglobulin (Ig)E and eosinophils in the development of AD boost inflammation and skin disruption through the production of oxidative stress, toxic granule proteins, cytokines, and chemokines [69]. Th2-related cytokines, such as IL-4, IL-5, IL-13, IL-22, and IL-31, are largely expressed in AD skin [70]. There are increasing reports of AD comorbidities, including neuropsychiatric, cardiovascular, and malignant disorders [71]. AD is the most common inflammatory skin disorder, affecting 10–25% of children and 2–10% of adults [72]. Recent investigations have illustrated the fundamental role of miRNA in AD pathogenesis (Figure 4) [73]. Elevated expressions of miR-10a, miR-24, miR-27a, miR-29b, miR-146a, miR-151a, miR-193a, miR-199, miR-211, miR-222, miR-4207, and miR-4529-3p were observed in the lesional skin of AD patients [25,74]. On the other hand, miR-135a, miR-143, miR-184, miR-194-5p, and miR-4454 were downregulated in clinical AD. miR-155-5p is also highly expressed in AD lesions, which can activate T cells, increase cutaneous inflammation, and disintegrate tight junctions [75]. miR-720 is upregulated in AD, possibly because of its role in keratinocyte cell cycle regulation [76]. The dysregulation of miR-143, miR-146a, miR-155, and miR-451a in AD can be used as a biomarker to diagnose this inflammatory disorder [77]. These miRNAs function in keratinocyte proliferation regulation, cytokine signaling, the NF-κB-dependent inflammation response, and T cell activation. Since plasma platelets are also involved in the pathogenesis of AD, recent studies [78] have indicated that platelet-associated miRNAs, such as miR-24 and miR-191, are responsible for the worsening of AD symptoms due to platelet activation.

Some miRNAs alleviate inflammation caused by AD through the suppression of the immune response in keratinocytes or immune cells. The mimics or agomirs of these miRNAs are potential candidates for anti-AD therapy. CCL22 is a macrophage-derived chemokine correlated with the severity of AD. Yoon et al. [79] assessed the suppression of the CCL22 gene by miRNA for treating AD in mice. A recombinant strain of *Salmonella typhimurium* expressing CCL22 miRNA (ST-miRCCL22) was prepared for CCL22 knockdown, and the successful transport of ST-miRCCL22 into the RAW264.7 macrophages was observed. The expression of CCL22 in the mouse splenocytes was reduced by about 10-fold after the ST-miRCCL22 treatment. In the in vivo study of atopic mice, the oral inoculation of ST-miRCCL22 lowered the total scratching counts for seven days. The numbers of IgE, IL-4, and Th17 cells were reduced after this treatment due to the CCL22 downregulation in the activated lymphocytes. miR-10a-5p has been acknowledged as a regulator of cell proliferation and inflammatory responses, and has been found to be upregulated in AD patients and in the proliferation of keratinocytes [80]. After the transfection of miR-10a-5p mimics into IL-1β-stimulated keratinocytes, IL-8 and CCL5 expression was significantly reduced. In the transfected cells, 48% were in the G1/G0 phase, compared with 38% for the untreated control, suggesting that proliferation was inhibited by the mimics. The luciferase assay verified that hyaluronan synthase 3, a positive regulator of keratinocyte proliferation and migration, is the direct target of miR-10a-5p.

Yang et al. [81] found a decreased expression of miR-124 in the lesional skin of AD patients compared with the non-lesional sites. The transfection of miR-124 mimics into keratinocytes elicited a 130-fold increase in miR-124 expression. This increase led to the downregulation of IL-8, CCL5, and CCL8 in the IFN-γ- or TNF-α-activated cells. RELA (the gene name of p65) is the direct target of miR-124 to control the NF-κB-associated inflammatory pathways in activated keratinocytes. IL-13 is a Th2-derived cytokine that can impair the epidermal barrier. IL-13Rα1 is a direct target of miR-143 [82]. IL-13 stimulation on keratinocytes resulted in a decrease in the miR-143 level [83]. The amount of IL-13Rα1 in the IL-13-stimulated keratinocytes was diminished by about 10-fold after transfection with the miR-143 mimics. The forced miR-143 expression prevented the IL-13-induced downregulation of filaggrin, loricrin, and involucrin. The skin barrier function was expected to be restored by this effect. In addition to psoriasis, miR-146a is applicable for AD treatment because of its involvement in immune regulation [84]. Meisgen et al. [85] transfected keratinocytes with synthetic miR-146a and found a remarkable suppression of the TLR2-induced production of TNF-α, IL-8, and CCL20. This downregulation was mediated by the direct targeting of miR-146a to TRAF6 and IRAK1. The transcriptomic analysis revealed that the miR-146a mimics regulated the genes involved in cell–cell communication, keratinocyte immunity, cytokines, chemokines, and antimicrobial peptides. miR-146a overexpression in the keratinocytes also lessened the chemotactic migration of neutrophils (0.54-fold as compared with the control). The evidence of miR-146a’s ability to alleviate skin inflammation in AD was further evaluated in vivo [86]. The AD-like model was established by the topical treatment of MC903, a vitamin D3 analog, on the ears of wild-type and miR-146a^−/−^ mice. The miR-146a-deficient mice developed a stronger inflammation response characterized by increased immune cell infiltration, as well as IFN-γ, CCL5, and CCL8 expression, in the skin. The keratinocyte-based study testified the direct targets of CARD10 and IRAK1 for miR-146a.

The lympho–epithelial kazal-type inhibitor (LEKTI) has relatively low expression in the keratinocytes of AD patients [87]. This decrease might have resulted from the overexpression of Yes-associated protein 1 (YAP1), a regulator of the proliferation of epidermal stem cells [88]. Cheng et al. [89] employed a luciferase reporter assay to approve the target binding of miR-375-3p with the 3′UTR of YAP1. The miR-375-3p expression was upregulated six-fold after the transfection of mimics in HaCaT cells. This upregulation contributed to the inhibition of IL-1β and IL-6, accompanied by a reduction of NF-κ nuclear translocation. The cell proliferation was also restrained by miR-375-3p transfection. miR-1294 has been recognized as a tumor suppressor. The role and regulatory mechanism of miR-1294 in AD were explored by Yan et al. [90]. In an in vitro 3D skin-equivalent model, the miR-1294 mimic treatment reduced the thickening of the lamellar bilayer structure stimulated with IFN-γ and TNF-α. The filaggrin level was reduced by IFN-γ and TNF-α was also recovered by the mimic. The in vivo dinitrochlorobenzene-induced AD-like mouse model exhibited a reduction of the injury score from three to one after the miR-1294 mimic treatment. miR-1294 upregulation decreased inflammation and skin barrier destruction by targeting STAT3 to inhibit NF-κB signaling. The targets and biological mechanisms of the anti-inflammatory miRNAs used to treat AD are summarized in Table 2.

### 3.3. Skin Wounds

Commonly observed cutaneous wounds include open wounds, infected wounds, diabetic wounds, burn wounds, and acne wounds. Wound healing is a complicated process consisting of four overlapping stages: hemostasis, inflammation, proliferation, and tissue remodeling. After the occurrence of a skin wound, numerous inflammatory cells migrate into the wound area to protect against microbial invasion and repair the damage. The dysregulation of inflammation generates unsuccessful healing, hypertrophic scarring, and keloids [91]. Appropriate inflammation is important for promoting skin wound healing. Nevertheless, redundant inflammation responses prompt pathological damage to wound tissue and delay repair [92]. miRNAs possess a strong potential to regulate both the induction and resolution of inflammation in skin wound healing [93]. The overexpression of miR-21, miR-29b, miR-106b, and miR-146a has been reported to accelerate re-epithelialization and reduce excessive scar generation in wound healing [94]. Contrary to this effect, the downregulation of miR-200c, miR-210, and miR155 is effective in improving wound healing. Because of the role of regulating inflammation and immunity, epidermal keratinocytes are the major cells participating in skin wound healing [95]. The re-epithelialization of the wound area by keratinocyte migration is an essential step of wound closure. Some miRNAs involved in psoriasis, such as miR-21, miR-31, and miR-203, are also implicated in keratinocyte migration [96]. Neutrophils are the primary immune cells in the early inflammatory response during wound repair [97]. The neutrophil-derived miR-142 is required to promote neutrophil migration and increase the ability of the wound site to resist microbial infection [98]. Macrophages are another type of immune cell governing the inflammatory phase during wound repair. miR-21 and miR-223 participate in the regulation of macrophage polarization in cutaneous wounds [99,100]. A clinical trial has investigated the effect of miR-29 mimics (Remlarsen) on skin wounds [101]. Intradermal miR-29 mimic injection into the incisional wound site reduced collagen expression and the development of fibroplasia accompanied by the downregulation of the miR-29 target genes *COL1A1*, *COL1A2*, and *COL3A1*.

The wound-healing process can be accelerated by treatment with anti-inflammatory miRNAs. Li et al. [102] signified the potential of miR-23b to inhibit inflammatory reactions in wound repair. miR-23b agomir transfection into HaCaT showed a wound closure of more than 90% ten days post-wounding in a scratch wound healing assay. Subcutaneous miR-23b injections into excisional wounds in mice decreased the immune cell accumulation and cytokine expression for accelerating healing. α-Smooth-muscle actin (α-SMA) in fibroblasts can secrete collagen for strengthening the wound [103]. miR-23b can promote the release of α-SMA in the fiber pattern. miR-23b inhibits inflammation by targeting apoptotic signal-regulating kinase 1 (ASK1). The miR-31 mimics were effective at enhancing wound healing via increased keratinocyte migration [104]. A surgical wound was created in healthy subjects. The basal level of miR-31 in the skin was low, but quickly increased by 1.9-fold one day after the injury. The miR-31 expression was continuously upregulated to 7.7-fold after seven days. miR-31 overexpression was induced using transforming growth factor (TGF)-β2. Epithelial membrane protein 1 (EMP-1) was the direct target of miR-31 in the keratinocytes. The in vitro scratch assay indicated that the miR-31 mimic increased the migration capability of the keratinocytes by 3.6-fold.

miR-34a is a tumor suppressor with the ability to regulate the immune response [105]. An excisional wound was made in mice to check the effect of miR-34a on wound closure [106]. miR-34a was downregulated in the inflammatory stage and returned to the baseline in the proliferative phase. The miR-34a^−/−^ mice showed impaired healing as compared with the wild-type animals. The re-epithelialization was faster in the group of wild-type mice than in the miR-34a-knockout mice (re-epithelialization percentage of 100% versus 10% after seven days). IL-6/STAT3 signaling was essential in the wound healing of the miR-34a^−/−^ mice. This result indicated the importance of miR-34a in inhibiting the inflammation of excisional wounds. Diabetic ulcers in the foot are the main comorbidity in diabetic patients. The anti-inflammatory property of miRNA could be applicable to accelerate the healing of diabetic wounds. Ban et al. [107] stated that miR-497 mimics could reduce the overexpression of TNF-α, IL-1β, and IL-6 in human dermal fibroblasts under hyperglycemic situations. The therapeutic efficacy of the mimics was investigated by intradermal injections into wounds in diabetic mice. A faster reduction of the wound area was observed in the group receiving miR-497 treatment, with healing of 66% as compared with the negative control group (23%) on day four. The levels of TNF-α, IL-1β, and IL-6 in the injury site decreased by more than 20% compared with the negative control. The targets and biological mechanisms of the anti-inflammatory miRNAs for cutaneous wound healing are summarized in Table 3.

### 3.4. Other Uses

Hosts with pathogenic infections usually undergo inflammation due to proinflammatory cytokine/chemokine bursts by immune cells [108]. Some anti-inflammatory miRNAs have the potential to treat microbe-stimulated inflammation in the skin [109]. *C. acnes* is reported to represent more than 30% of the facial microbes in acne patients [110]. miR-146a has been successfully used to repress biofilm-derived *C. acnes*-triggered inflammation [111]. The overexpression of miR-146a by mimic transfection to keratinocytes showed markedly reduced TLR2-induced TNF-α, IL-6, and IL-8 expression. The data of the luciferase reporter assay suggested that miR-146a bound to the 3′UTR of IRAK1 and TRAF6, resulting in the inhibition of the ERK1/2, NF-κB, and mitogen-activated protein kinase (MAPK) pathways. *Candida* species are the most common fungal pathogens evoking skin and system infection. Dectin-1 is a significant sensor for β-glucan from *Candida* [112]. Dectin-1 and β-glucan can trigger the intracellular transduction pathways of CARD. CARD10 is the direct target of miR-146a. Du et al. [113] appraised the effect of miR-146a on the inflammation induced by *Candida albicans*. The transfection of miR-146a into *C. albicans*-stimulated THP-1 cells significantly inhibited Dectin-1-elicited TNF-α and IL-6 production by about two-fold. The miR-146a mimic inhibited the *C. albicans*-induced translocation of NF-κB.

Lupus erythematosus is an autoimmune disorder with a wide range of dermatological manifestations. Lupus erythematosus lesions in the skin share extensive lymphocyte infiltration with a high predominance of CD4 T cells and cytokines, including TNF-α, IL-1α, IL-1β, IL-6, and IL-8 [114]. Huang et al. [115] found a correlation between the increase in Th17 cells and the decrease in miR-590-3p in systemic lupus erythematosus patients and MRL/lpr mice. miR-590-3p agomir transfection promoted the apoptosis of Th17 cells by autophagy suppression via direct targeting of autophagy-related 7 (Atg7). The in vivo treatment of lupus mice using agomir lessened lupus nephritis and the size of skin lesions. Chronic idiopathic urticaria (CIU) is a polyetiological dermatological inflammation disorder. A total of 16 miRNAs were found to be differentially expressed in patients with CIU [116]. Among them, five miRNAs (29c-5p, 361-3p, 2355-3p, 2355-5p, and 4264) were largely increased in CIU, making them potential biomarkers for diagnosing autoimmune urticaria. miRNAs are active in the cell regulation of CIU. The CIU patients showed lower expression of miR-194 and higher thrombospondin 1 (THBS1) as compared with the healthy control [117]. THBS1 was proven to be the target of miR-194 in the luciferase activity assay in 293T cells. miR-194 mimics decreased the amount of TNF-α, IL-1β, IL-6, and IL-8 in mast cells. The mast cell degranulation and histamine release were also lowered by transfection with the mimic.

## 4. miRNAs as the Targets to Inhibit Skin Inflammation

Besides the capability of miRNAs to directly block mRNA activity and inhibit inflammation, miRNAs can be a target to mediate the anti-inflammatory response [118]. Some chemicals are able to target and modulate miRNAs to attain the aim of inflammation suppression. It has been proposed that miRNAs could be a group of biological target molecules for therapeutic intention. The application of compounds that target pri-miRNAs, pre-miRNAs, miRNA processing, and loading into the RISC structure has potential for drug design and development. Some bioactive molecules may impact endogenous miRNA synthesis through downregulation or upregulation, thereby contributing to inflammation suppression [119]. For instance, resveratrol, from the stilbene group, is considered to be beneficial for skin health. This polyphenol has been broadly reported as a potential molecule to treat various skin disorders, including skin cancer, photoaging, allergy, dermatitis, melanogenesis, and microbial infections [120]. Wang and Zhang [121] demonstrated the upregulation of miR-17 by resveratrol for reducing lipopolysaccharide-induced skin inflammation. The resveratrol intervention inhibited the production of TNF-α, IL-6, and IL-8 in the lipopolysaccharide-activated HaCaT. miR-17 was upregulated three-fold after resveratrol treatment. miR-17 silencing enhanced the expression of cytokines in the resveratrol-treated lipopolysaccharide-activated cells. The resveratrol–miR-17 axis was found to stimulate the phosphatase and tensin homolog (PTEN)/phosphoinositide 3-kinases (PI3Ks)/AKT and mammalian target of rapamycin (mTOR) pathways.

Adalimumab (Humira) is a biological drug used to ameliorate psoriatic inflammation via TNF inhibition [122]. In a clinical trial of psoriasis patients [123], adalimumab treatment was found to significantly decrease the psoriasis area and severity score (PASI) and miR-146a-5p in peripheral blood mononuclear cells (PBMCs). The reduction of the miR-146a-5p levels was correlated with the improvement of the PASI. miR-146a-5p could be a dynamic biomarker to predict the therapeutic effectiveness of adalimumab. Ebosin is an exopolysaccharide isolated from fermentation cultures of *Streptomyces* sp. 139. This compound could mitigate lipopolysaccharide-activated inflammation in HaCaT via IκB kinase (IKK)/NF-κB signaling [124]. Moreover, the PASI score of the imiquimod-treated psoriasiform mice was decreased from 3 to 1.5 with the application of ebosin. Ebosin reduced inflammation by lessening miR-155-3p expression, both in vitro and in vivo. The luciferase activity assay indicated the direct targeting of TNF alpha-induced protein 3 (TNFAIP3) by miR-155-3p. Circular RNA (circRNA) is a type of lncRNA. CircRNA has no 5′ or 3′ end, which endows it with resistance to exonuclease [125]. CircRNA RAB3B, a member of the RAS oncogene family, has been found to be downregulated in psoriasis [126]. CircRAB3B overexpression delayed the proliferation and elevated the apoptotic rate of IL-22-stimulated HaCaT [127]. miR-1228-3p was the target of circRAB3B, and this circRNA negatively regulated the expression of miR-1228-3p in keratinocytes. The luciferase reporter and bioinformatic analyses showed the direct binding of miR-1228-3p to the 3′UTR of PTEN. The combination of 8-methoxypsoralen and UVA (PUVA) is an effective photochemotherapy used to treat psoriasis. Chowdhari and Saini [128] detected significant upregulation of has-miR-4516 in the HaCaT after PUVA application. The transfection of has-miR-4516 mimics decreased STAT3 and pSTAT3 by 1.5-fold in HaCaT cells. The overexpression of has-miR-4516 raised the content of apoptotic keratinocytes from 4% to 24%.

Some bioactives are capable of directly modulating the expression of miRNAs for alleviating inflammatory dermatitis. Berberine is a natural alkaloid derived from *Coptis chinensis*. This bioactive compound has been proven to show anti-inflammatory, antioxidant, anticancer, and hypolipidemic effects [129]. Berberine attenuated ear swelling from 0.48 to 0.33 mm in a mouse model of allergic dermatitis [130]. This treatment also inhibited miR-21 expression, histamine release, and p38 phosphorylation. The result of the miR-21 mimic transfection in the mast cells indicated that miR-21-mediated suppression in mast cell degranulation was involved in the anti-inflammatory activity of berberine in dermatitis.

Acupuncture, originating from ancient China, involves inserting needles into the body to stimulate sensory nerves in the skin and muscles. Electro-acupuncture is an improvement of traditional acupuncture by the addition of an electrical charge, promoting needle stimulation through electrical impulses [131]. Wang et al. [132] used electro-acupuncture to reduce the inflammation caused by allergic dermatitis. Treatment with electro-acupuncture at the ST36 acupoint resulted in the reduction of the ear thickness from 0.3 to 0.2 mm in allergic dermatitis-like rats. The acupuncture treatment lowered the expression of miR-155 through the signaling of IL-33 for inhibiting p38 phosphorylation. The rat peritoneal mast cells transfected with miR-155 mimics abrogated the inhibitory effect of electro-acupuncture on NF-κB-regulated transcription in response to IL-33. IL-32γ is an anti-inflammatory cytokine that inhibits skin inflammation [133]. Lee et al. [134] proved that the AD severity and epidermal thickness of MC903-induced IL-32γ transgenic mice were lower than those of wild-type animals. The expression of miR-205 was impeded by IL-32γ in the mouse skin and HaCaT cells. The expression of TNF-α, IL-1β, IL-6, and thymic stromal lymphopoietin (TSLP) in IFN-γ/TNF-α-activated keratinocytes. Belinostat is a histone deacetylase inhibitor used for the suppression of hematological and solid malignancies. It could potentially target miR-335 to restore barrier defects in AD [135]. The luciferase reporter analysis confirmed the direct binding of miR-335 to SOX6 3′UTR. The miR-335 level was aberrantly lost in the lesional skin of AD patients. In an ex vivo human organ culture model mimicking the AD phenotype, topical applications of belinostat upregulated filaggrin and involucrin, the downstream of miR-335. Thus, the barrier function of AD-like skin could be recovered by this effect.

Cutaneous wound healing can be accelerated by chemicals such as vitamin D and resveratrol. In addition, some natural extracts are beneficial for treating skin wounds. Ginger has been proven to resolve the problem of poor wound healing [136]. Al-Rawaf et al. [137] combined vitamin D and ginger supplements to treat diabetic wounds in rats. The combined treatment in the diabetic wounds accelerated the epithelialization period from 18.8 to 11.3 days. Compared with healthy rats, diabetic rats exhibited greater levels of miR-155 and lower levels of miR-15a and miR-146a. The combined vitamin D and ginger treatment significantly reversed this tendency. Resveratrol has been shown to be favorable for promoting skin wound repair [138]. Hu et al. [139] discovered the beneficial effect of resveratrol on diabetic wounds by raising the expression of extracellular vesicle (EV)-carried miRNA-129 derived from mesenchymal stem cells (MSCs). Rat MSCs were isolated and treated with resveratrol, and the corresponding EVs were isolated to promote skin wound healing. The size distribution of the EVs ranged between 40 and 150 nm, indicating a nano size. More than 80% of human umbilical-vein endothelial cells (HUVEC) showed internal uptake of EVs based on fluorescence microscopy. The use of resveratrol-treated EVs in diabetic wounds improved the proliferative and migratory potential of the cells. Resveratrol promoted wound healing through TRAF downregulation via MSC-EV-carried miR-219. Curcumin is known to improve diabetic wound repair [140]; however, its low bioavailability and poor aqueous solubility have prevented the clinical application of curcumin. Huang et al. [141] employed (2*E*,6*E*)-2,6-bis(2-(trifluoromethyl)benzylidene)cyclohexanone (C66), a synthetic analog of curcumin, to resolve these problems. The C66 treatments in diabetic wounds showed complete closure within 14 days, whereas the non-treatment control had a low closure rate of 64%. The decreased miR-146a level in the diabetic wound was upregulated after C66 treatment. The C66 administration also showed pronounced inhibition of the expression of TNF-α, IL-6, and IL-8. The cell-based study indicated that C66 reversed NF-κB activation due to the overexpressed miR146a in HUVECs.

Jiang Tang Xiao Ke (JTXK) is a traditional Chinese formula containing extracts of pueraria, rehmannia, ginseng, and radix salvia miltiorrhizae. This medicine has been reported to decrease miR-139-5p expression in the pancreatic tissue of diabetic mice [142]. To evaluate the potential of JTXK on *S. aureus*-infected wound healing, a topical noisome hydrogel was utilized to load JTXK [143]. The in vivo data supported the decreased miR-139-5p expression in the infected wound after topical JTXK administration and the accelerated wound healing rate. Eif4g2, the key downstream mediator of miR-139-5p, was significantly increased by about two-fold by JTXK treatment. *Staphylococcus epidermidis* plays a vital role in controlling the skin inflammation response. Lipoteichoic acid released from *S. epidermidis* inhibits *C. acnes*-mediated inflammation in the skin [144]. Lipoteichoic acid activated TLR2 to upregulate miR-143. This miRNA, in turn, targeted TLR2 to decrease the stability of the TLR2 mRNA and then lessen the TLR2 proteins, thus suppressing the proinflammatory cytokines induced by *C. acnes*. *C. acnes*-bearing mice treated with lipoteichoic acid exhibited decreased erythema and ear swelling as compared with the control group. UVB irradiation generates skin photoaging by the induction of cell death and DNA damage. Lee et al. [145] showed the protective activity of troxerutin on UVB-elicited photoaging in keratinocytes. Troxerutin is a natural flavonoid with anti-inflammatory and antioxidant characteristics [146]. An eight-hour pretreatment with 5 μM of troxerutin increased the UVB-irradiated keratinocyte viability by 20%. miRNA gene microarray analysis showed that 68 miRNAs were modulated after troxerutin treatment of UVB-exposed keratinocytes. Among them, the miR-205-3p expression was elevated by 4.3-fold, while miR-483-5p, miR-513b, and miR-3648 were decreased by 16.6-, 23.1-, and 11.6-fold, respectively. Based on these data, the protective effect of troxerutin could be grouped into four functions: apoptosis, proliferation, migration, and DNA repair. The miRNA targets and biological mechanisms of the bioactive molecules for inhibiting skin inflammation in different skin disease models are summarized in Table 4.

## 5. Approaches for Improving miRNA Delivery

Topical administration routes provide a direct way to treat skin inflammation. Topical drug delivery is a noninvasive and convenient strategy for treating cutaneous disorders. It has the advantages of direct access to the nidus, minimal off-target effects, and the avoidance of systemic responses [147]. miRNA administration via topical absorption can be an ideal approach for applying therapies to the skin [96]. However, the intrinsic barrier function of the stratum corneum, combined with the hydrophilic features of miRNA, has precluded the successful permeation of miRNA into the skin. Even if miRNA can penetrate the inflamed skin, miRNA-mediated gene regulation still requires an intracellular entrance into the target cells. Unfortunately, it is difficult for naked miRNA to permeate the skin and the cell membrane. A delivery system is, therefore, required for facile miRNA administration [148]. Effective topical therapies using miRNA require bypassing the skin barrier and the subsequent miRNA transfection into the target cells. The necessity of using carriers to enhance miRNA delivery is urgent to achieve extensive application in skin inflammation treatments.

Cell-penetrating peptides (CPPs) are one of the strategies for enhancing miRNA penetration into cells and the skin [149]. CPPs are short peptides (<30 amino acids) capable of translocating themselves into cells and facilitating cargo or CPP/cargo complexes to translocate across the plasma membrane [150]. The skin-permeation of bioactives can be improved by using CPPs as penetration enhancers [151]. Urgard et al. [152] used the CPP PepFect6 to form a nanocomplex with miR-146a for treating irritant dermatitis. The nanocomplex exhibited a spherical and homogeneous particle distribution with an average diameter of 30–50 nm. The facile internalization of the miR-146a mimic/PepFect6 nanocomplex into the keratinocytes led to the suppression of the direct targets CARD10 (1.8-fold) and IRAK1 (2.2-fold). In a mouse model of irritant contact dermatitis, the administration of the nanocomplex increased the miR-146a expression by 1380-fold after 30 h. The ear swelling was attenuated 2.4-fold after nanocomplex application. Mulholland et al. [153] developed miRNA-31/CPP nanocarriers within an electrospun nanofiber, with the aim of regenerating skin wounds. The CPP used in this nanocomplex was CHAT, which is a 15-amino-acid linear peptide considered useful for enhancing plasmid DNA delivery [154]. The prepared nanocomplex had a mean size and zeta potential of 74 nm and 9.7 mV, respectively. The transfection percentage of the nanocomplex to the HaCaT cells was greater than 40%. The electrospun nanofiber was advantageous for wound healing due to its biocompatibility and close skin coverage [155]. In vivo, topical treatments of the nanocomplex-loaded electrospun nanofiber on the mouse wound increased the epidermal thickness and angiogenesis as compared with the commercial dressing control.

Nanoparticles are promising delivery systems that could ameliorate the cellular uptake of miRNA. The use of nanoparticles protects miRNA from degradation and improves the efficiency of delivery. Zgheib et al. [156] conjugated miR-146a with cerium oxide nanoparticles for accelerating diabetic wound repair. By scavenging reactive oxygen species (ROS), this type of nanoparticle could eliminate oxidative stress and regulate the imbalance between oxidant and antioxidant enzymes in diabetic wounds [157]. The hydrodynamic diameter of the miR-146a-conjugated nanoparticles was approximately 190 nm. Diabetic wounds were induced by injecting streptozocin into a pig. After a 10-day application, the wound surface area of the nanoparticle group (4.8 cm^2^) was significantly smaller than that of the control (6.8 cm^2^). The wound was completely closed on days 14 and 18 after the nanoparticle and saline treatments, respectively. Niemiec et al. [158] further incorporated miR-146a-conjugated nanoparticles into silk fibroin to improve diabetic wound repair. Silk fibroin, composed of biocompatible polymers, is characterized by a strong mechanical structure and the ability to exhibit strain hardening [159]. In the murine model of diabetic wounds, the wounds treated with nanoparticle-incorporated nanosilk and saline were reduced to 31% and 8% of the original size after 13 days, respectively. The human skin samples treated with nanosilk had increased biomechanical strength (51 N) compared with the saline control (42 N). The proinflammatories IL-6 and IL-8 in the wound site were also reduced by the nanosilk application.

Amphipathic bile acid-attached polyethyleneimine (BA-PEI) imparts facile cell membrane permeability by membrane fusion and pore creation [160]. Wang et al. [161] fabricated BA-PEI nanocarriers to load synthetic miR-21 and accelerate excisional wound healing. The nanosystem displayed a size of 173 nm with a zeta potential of 27 mV. An 83-fold increase in miR-21 expression was observed after the treatment with the nanocarriers in HaCaT compared with the saline control. Subcutaneous injections of the nanocarriers in the wound sites of the wild-type mice showed a 57% open wound area after eight days, whereas a 100% open wound area was detected for the group receiving the saline treatment. The nanocarrier-treated wound was fully closed on day 16. There was an open wound and scar formation in the saline group on day 16. Saleh et al. [162] developed bioadhesive hydrogels incorporated with miR-223-5p-loaded hyaluronic acid nanoparticles to control macrophage polarization during wound healing. The hydrogels were composed of gelatin methacryloyl because of its robust attachment to the wound [163]. The miR-223-5p-loaded nanoparticles exhibited a mean diameter of 160 nm and a surface charge of −13 mV. The amount of miR-223-5p in the M1 macrophages was increased by 1541-fold after 24 h of nanoparticle incubation. A murine excisional wound model demonstrated a greater wound closure percentage due to the nanoparticle-laden hydrogels (96%) compared with the naked miRNA (67%), hydrogels (61%), and non-treatment control (45%). The collagen level and epidermal thickness in the wound site were also significantly increased by the nanoparticle-laden hydrogels. Feng et al. [164] prepared biomimetic reconstituted high-density lipoprotein nanogels loaded with miR-210 antisense to explore the anti-inflammatory effect on imiquimod-induced psoriasiform lesions in mice. The average size of the nanocarriers was about 30 nm. Topical application of the nanogels significantly reduced the erythema, scales, and immune cell accumulation in the lesions. The proportion of Th1 and Th17 cells in the lesional skin was decreased by this treatment and was accompanied by decreased IFN-γ and IL-17A.

Exosomes are membrane-enclosed nanovesicles released by cells into extracellular spaces or culture mediums for managing cell–cell communication [165]. Genetic materials, lipids, and proteins are contained inside the exosomes. Because of their biomimetic features and targeting capabilities, exosomes can be used as nanocarriers for drug delivery [166]. Xia et al. [167] designed an exosome-guided cell technique with miRNA-125b transfection to elicit cutaneous wound healing. Fibroblast activation to myofibroblasts can alleviate age-related defects in wound repair. Supplementing wounds with exosomes isolated from young mouse wound-edge fibroblasts largely improved the myofibroblast abundance in the aged mice and promoted fibroblast transition to the myofibroblasts, thus accelerating wound closure. The exosomal transfer of miR-125b to the fibroblasts suppressed sirtuin 7, the direct target of muR-125b, to accelerate myofibroblast differentiation. The replenishment of miR-125b could be a therapeutic strategy to enhance wound repair. To prepare the nanocarriers as gene-delivery systems, soluble potato starch was reacted with a quaternization reagent to produce quaternized starch (Q-starch) [168]. This nanosystem, based on natural polysaccharides, was considered useful as a drug delivery carrier due to its biodegradability, minimal immunogenicity, and possible receptor-mediated endocytosis [169]. Lifshiz Zimon et al. [170] assessed the benefits of ultrasound-assisted miR-197/Q-starch nanocomplexes for improving skin absorption as well as its anti-psoriatic activity. The ability of low-frequency ultrasound to enhance cell membrane permeability and skin delivery was elucidated previously [171]. The mean diameter of the nanocomplex was estimated to be 132 nm, with a zeta potential of 32 mV. The ultrasound-mediated delivery contributed to the entrance of the nanocomplex to the epidermis, including the basal cells. The in vivo efficacy of the ultrasound-mediated nanocomplex absorption was evaluated by the xenograft transplantation of human psoriasis skin to the mice. The pathological score data showed a reduction after the topical application of the nanocomplex in the presence of ultrasound. The epidermal hyperplasia was also restrained by the combined ultrasound and nanocarriers. The approaches for improving miRNA delivery into cells and skin are depicted in Table 5.

## 6. Conclusions

The regulation of miRNA expression is a promising and novel therapy for targeting skin inflammation diseases, such as psoriasis, AD, and cutaneous wounds. The exogenous administration of the anti-inflammatory miRNA mimic is beneficial for inhibiting proinflammatory mediators, leading to the alleviation of skin inflammation. miRNA-based anti-inflammatory therapy is also achieved by treatment with bioactive agents that can modulate the expression of miRNA. In terms of using miRNA treatment for skin diseases, local administration via the skin could be an efficient approach to achieve satisfactory availability. Topical delivery of miRNA usually has an incomplete response. This phenomenon is mainly caused by the barrier features of the skin and the target cells. Hence, the elaboration of delivery carriers that improve skin delivery and cell internalization is important. Considering the efficiency of skin penetration and controlled release, the introduction of nanocarriers could be a potential solution for topical application. Regarding future applications, effort should be paid to connecting the gap between laboratory investigations and clinical trials. Most studies on the anti-inflammatory activity of miRNA have been conducted using cell- and animal-based models, and there have been few clinical studies until now. The high cost of miRNA synthesis and its questionable stability may hinder the progress of its application. Although miRNA-based therapies have some limitations, future approaches aimed at treating cutaneous inflammation in a variety of skin diseases should be considered.

## Figures and Tables

**Table 1:** The targets and biological mechanisms of anti-inflammatory miRNAs for treating psoriasis.

miRNA Code	Targets	Experimental Models	Outcome	Reference
miR-99a	FZD5 and FZD8	HaCaT cells and patients	Keratinocyte proliferation inhibition through β-catenin signaling	Shen et al. [47]
miR-125a	CAMK4	HaCaT cells and patients	Keratinocyte proliferation inhibition and apoptosis enhancement	Su et al. [49]
miR-146a	CARD10, FERMT1, IRAK1 and TRAF6	miR-146a^−/−^ and wild-type mice and patients	Inhibited psoriasiform inflammation, hyperplasia, and neutrophil infiltration	Srivastava et al. [51]
miR-146b	CARD10, FERMT1, IRAK1 and TRAF6	Normal human epidermal keratinocytes and miR-146a^−/−^ or miR-146b^−/−^ mice	Modulation of inflammatory response and keratinocyte proliferation	Hermann et al. [53]
miR-146a/b	CARD10, FERMT1, IRAK1 and TRAF6	Normal human epidermal keratinocytes	SERPINB2 is coordinately regulated in the psoriatic inflammation with miR-146a/b	Vaher et al. [55]
miR-187	CD276	HaCaT, wild-type mice, and patients	Inhibition of keratinocyte hyperproliferation	Tang et al. [56]
miR-193b-3p	ERBB4	HaCaT and wild-type mice	Blockade of psoriasis-like inflammation through NF-κB/STAT3 signaling	Huang et al. [58]
miR-203a	Kynureninase	HaCaT and wild-type mice	Reduction of IL-1β in cytokine-activated keratinocytes	Wang et al. [60]
miR-214-3p	FOXM1	HaCaT, wild-type mice, and patients	Inhibition of keratinocyte hyperproliferation and psoriasiform inflammation	Zhao et al. [62]
miR-215-5p	DYRK1A	HaCaT and wild-type mice	Suppression of proliferation and cell cycle progression of keratinocytes	Liu et al. [63]
miR-340	IL-17A	293T cells and wild-type mice	Reduction of psoriasiform symptoms	Bian et al. [64]
miR-383	LCN2	Cells from the skin of imiquimod-treated rats	Reduced cell proliferation and increased cell apoptosis	Wang et al. [66]
miR-489-3p	TLR4	HaCaT	Inhibition of keratinocyte proliferation and TLR4/NF-κB signaling	Ye et al. [67]

CAMK4, calmodulin-dependent protein kinase IV; CARD10, caspase recruitment domain family member 10; CD276, cluster of differentiation 276; DYRK1A, dual-specificity tyrosine phosphorylation regulated kinase 1A; ERBB4, Erb-B2 receptor tyrosine kinase 4; FERMT1, fermitin family homolog 1; FOXM1, forkhead box M1; FZD, frizzled; IRAK1, IL-1 receptor-associated kinase 1; LCN2, lipocalin 2; NF-κB, nuclear factor-κB; STAT3, signal transducer and activator of transcription 3; TLR, Toll-like receptor; TRAF6, tumor necrosis factor receptor-associated factor 6.

**Table 2:** The targets and biological mechanisms of anti-inflammatory miRNAs for treating atopic dermatitis.

miRNA Code	Targets	Experimental Models	Outcome	Reference
ST-miRCCL22	CCL22	RAW264.7 macrophages and wild-type mice	Reduction of IgE, IL-4, and Th17 cells	Yoon et al. [79]
miR-10a-5p	Hyaluronan synthase 3	Keratinocytes and patients	Inhibition of keratinocyte proliferation and cytokines/chemokines	Vaher et al. [80]
miR-124	RELA (the gene name of p65)	Keratinocytes and patients	Downregulation of IL-8, CCL5, and CCL8	Yang et al. [81]
miR-143	IL-13Rα1	Keratinocytes	Enhancement of the synthesis of filaggrin, loricrin, and involucrin	Zeng et al. [83]
miR-146a	IRAK1 and TRAF6	Keratinocytes	Suppression of TLR2-induced production of TNF-α, IL-8, and CCL20.	Meisgen et al. [85]
miR-146a	CARD10 and IRAK1	Keratinocytes and wild-type and miR-146a^−/−^ mice	Alleviation of chronic skin inflammation through innate immune response suppression in keratinocytes	Rebane et al. [86]
miR-375-3p	YAP1	HaCaT	Inhibition of IL-1β and IL-6 accompanied by a reduction in NF-κ nuclear translocation	Cheng et al. [89]
miR-1294	STAT3	HaCaT, 3D skin equivalent, and wild-type mice	Decrease in inflammation and skin barrier destruction	Chen et al. [90]

CARD10, caspase recruitment domain family member 10; CCL, chemokine (C-C motif) ligand; IgE, immunoglobulin E; IL, interleukin; IRAK1, IL-1 receptor-associated kinase 1; STAT3, signal transducer and activator of transcription 3; ST, *Salmonella typhimurium*; TLR, Toll-like receptor; TNF-α, tumor necrosis factor-α; TRAF6, tumor necrosis factor receptor-associated factor 6; YAP1, Yes-associated protein 1.

**Table 3:** The targets and biological mechanisms of anti-inflammatory miRNAs for skin wound healing.

miRNA Code	Targets	Experimental Models	Outcome	Reference
miR-23b	ASK1	HaCaT and wild-type mice	Inhibition of cytokines and enhancement of α-SMA expression	Li et al. [102]
miR-31	EMP-1	Keratinocytes and healthy volunteers	Enhancement of wound healing via increased keratinocyte migration	Li et al. [104]
miR-34a	Bcl-2 and CCND1	Wild-type and miR-34a^−/−^ mice	miR-34a deficiency leads to impaired wound closure	Zhao et al. [106]
miR-497	AKT2 and E2F3	Human dermal fibroblasts and wild-type mice	Inhibition of cytokines and acceleration of diabetic wound healing	Ban et al. [107]

AKT2, RAC-β serine/threonine-protein kinase; ASK1, apoptotic signal-regulating kinase 1; α-SMA, α-smooth-muscle actin; Bcl-2, B-cell lymphoma 2; CCND1, cyclin D1; E2F3, E2F transcription factor 3; EMP-1, epithelial membrane protein 1.

**Table 4:** miRNAs as the targets of bioactive molecules for inhibiting skin inflammation.

Bioactive Molecule	Target miRNAs	Experimental Models	Outcome	Reference
Resveratrol	miR-17	HaCaT	Resveratrol upregulates miR-17 for alleviated lipopolysaccharide-induced inflammation	Wang and Zhang [121]
Adalimumab	miR-146a-5p	Psoriasis patients	Reduction of miR-146a-5p is associated with the improvement of psoriasis	Mensà et al. [123]
Ebosin	miR-155-3p	HaCaT and wild-type mice	Ebosin reduces psoriatic inflammation through miR-155-3p/IL-17 axis	Guo et al. [124]
CircRAB3B	miR-1228-3p	HaCaT	CircRAB3B negatively regulates the expression of miR-1228-3p	Lu et al. [127]
PUVA	hsa-miR-4516	HaCaT	has-miR-4516 mediates PUVA-induced apoptosis in keratinocytes	Chowdhari and Saini [128]
Berberine	miR-21	Mast cells and wild-type mice	Berberine mitigates allergic dermatitis via miRNA/p38 signaling	Li et al. [130]
Electro-acupuncture	miR-155	Mast cells and wild-type rats	Acupuncture lowered the expression of miR-155 through the signaling of IL-33	Wang et al. [132]
IL-32γ	miR-205	HaCaT and wild-type and IL-32γ transgenic mice	IL-32γ inhibited AD through downregulation of miR-205	Lee et al. [134]
Belinostat	miR-335	Keratinocytes and AD patients		Liew et al. [135]
Vitamin D and ginger	miR-15a, miR-146a, and miR-155	Wild-type rats	Combined treatment of vitamin D and ginger decreased miR-155 and increased miR-15a and miR-146a	Al-Rawaf et al. [137]
Resveratrol	miR-129	HUVEC and wild-type rats	Resveratrol promoted wound healing through TRAF downregulation via MSC-EV-carried miR-219	Hu et al. [139]
C66	miR-146a	HUVEC and wild-type mice	Decreased miR-146a level in diabetic wounds was upregulated after C66 treatment.	Huang et al. [141]
Jiang Tang Xiao Ke	miR-139-5p	Wild-type and miR-139^−/−^ mice	Decreased miR-139-5p expression in the infected wound after topical JTXK administration	Zhang et al. [143]
Lipoteichoic acid	miR-143	Keratinocytes and wild-type mice	Lipoteichoic acid activated TLR2 to upregulate miR-143	Xia et al. [144]
Troxerutin	miR-205-3p, miR-483-5p, miR-513b, and miR-3648	HaCaT	miR-205-3p expression was elevated, while miR-483-5p, miR-513b, and miR-3648 expressions were decreased by troxerutin	Lee et al. [145]

AD, atopic dermatitis; C66, (2*E*,6*E*)-2,6-bis(2-(trifluoromethyl)benzylidene)cyclohexanone; HUVEC, human umbilical vein endothelial cell; PUVA, psoralen and ultraviolet A; TLR, Toll-like receptor.

**Table 5:** The approaches for facile delivery of miRNAs into target cells and skin.

miRNA Code	Approach	Inflammation Models	Outcome	Reference
miR-146a	CPPs	Irritant contact dermatitis	Facile internalization of miR-146a/CPP nanocomplex into keratinocytes inhibits inflammation response	Urgard et al. [152]
miR-31	CPPs	Excisional wound	miRNA-31/CPP nanocomplex within an electrospun nanofiber facilely regenerates wounds	Mulholland et al. [153]
miR-146a	Cerium oxide nanoparticles	Diabetic wound	miR-146a-conjugated nanoparticles correct wound-healing impairment	Zgheib et al. [156]
miR-146a	Cerium oxide nanoparticles in silk fibroin	Diabetic wound	Incorporation of miR-146a-conjugated nanoparticles into silk fibroin improves the diabetic wound repair	Niemiec et al. [158]
miR-21	BA-PEI nanoparticles	Excisional wound	BA-PEI nanoparticles enhance the effect of miR-21 on wound healing	Wang et al. [161]
miR-223-5p	Hyaluronic acid nanoparticles in hydrogels	Excisional wound	Nanoparticle-loaded hydrogels control macrophage polarization during wound healing	Saleh et al. [162]
miR-210 antisense	Reconstituted high-density lipoprotein nanogels	Psoriasiform lesion	Topical application of the nanogels significantly reduces immune cell accumulation in lesions	Feng et al. [164]
miR-125b	Exosomes	Excisional wound	Exosomal transfer of miR-125b to fibroblasts suppresses sirtuin 7 to accelerate wound healing	Xia et al. [167]
miR-197	Ultrasound-mediated nanocomplex delivery	Xenograft transplantation mice	Ultrasound-assisted delivery enhances miR-197-loaded nanocomplex	Lifshiz Zimon et al. [170]

BA-PEI, bile acid-attached polyethyleneimine; CPPs, cell-penetrating peptides.
